# Micro-Photographs in Histology, Normal and Pathological

**Published:** 1877-07

**Authors:** 


### BIBLIOGRAPHICAL.

Micro-Photographs in Histology, Normal and Pathological. By Carl Seiler, M.D., in conjunction with J. Gibbons Hunt, M.D., and Joseph G. Richardson, M.D. Philadelphia, 1877.

This is the ninth number of the first volume of this very estimable publication. We find in it, first, a magnified photograph of a section of lung tissue; and, considering the difficulty of accurately defining, in a photograph, a distinct representation of this tissue, we think the work has been creditably performed. Three other plates are embraced in this issue: a section of tubercular lung, transverse section of a bronchial tubule, and air-tubes of silk worm. All the specimens from which these plates are taken are gotten up in the most approved style, and may be accepted as accurate representations.

